# Elevating Jak-STAT signaling via SOCS3 deletion sustains photoreceptor viability and visual function in mouse models of retinitis pigmentosa

**DOI:** 10.21203/rs.3.rs-7089882/v1

**Published:** 2025-07-11

**Authors:** Yanjie Wang, Steven Nusinowitz, Xian-Jie Yang

**Affiliations:** University of California, Los Angeles; University of California, Los Angeles; University of California, Los Angeles

**Keywords:** Retinal degeneration, CNTF, SOCS3, STAT3, Mouse models

## Abstract

Retinitis pigmentosa (RP) is an inherited retinal disease in which the loss of rod photoreceptors precedes cone photoreceptor degeneration. The neurocytokine ciliary neurotrophic factor (CNTF) can provide potent neuroprotection for photoreceptors in various retinal degeneration models and has thus been tested in clinical trials aimed at treating blinding diseases. In a preclinical model of RP, exogenous CNTF signaling mediated by the cytokine receptor gp130 initially triggered STAT3 and ERK phosphorylation in Muller glial cells and subsequently activated STAT3 in rods to promote photoreceptor survival. However, despite enhancing photoreceptor viability, the constitutive expression of exogenous CNTF perturbs the retinal transcriptome and further suppresses visual function. Activated STAT3 upregulates suppressor of cytokine signaling 3 (SOCS3), which acts as a feedback inhibitor to dampen cytokine signaling. In this study, we investigated whether eliminating SOCS3 in rod cells is sufficient to increase endogenous STAT3 signaling and enhance photoreceptor viability without exogenous CNTF. We show that rod-specific SOCS3 deletion attenuates photoreceptor degeneration and improves cone cell morphology in both the Pde6b/rd10 and Prph2(P216L)/rds mouse models of RP. SOCS3 ablation in rods not only causes STAT3 activation in rod photoreceptors but also leads to the propagation of STAT3 and ERK signaling to inner retinal cell types. Furthermore, rod SOCS3 deficiency led to improved visual function in the Pde6b/rd10 model and sustained cone function in the Prph2(P216L)/rds retina. Together, these findings demonstrate that intercellular communication occurs among retinal cells and the modulation of endogenous cytokine signaling events can be leveraged as an efficacious treatment to attenuate neuronal loss and preserve visual function.

## INTRODUCTION

Retinitis pigmentosa (RP) is a group of inherited eye diseases that affects 1 in 4000 people worldwide (1). At the early stage of RP, rod photoreceptors degenerate, and patients first exhibit night blindness with a loss of peripheral vision. With the progression of the disease, cone cells are affected, and RP patients develop impaired daytime color vision and eventually the loss of central vision. Several major types of RP can be distinguished by their pattern of inheritance as autosomal dominant, autosomal recessive, or X-linked. Most mutations causing RP are in genes expressed by photoreceptors. To date, more than one hundred genes and loci are associated with retinal degenerative diseases, including RP (2, 3). However, approximately 50% of RP cases are isolated without a previous family history (4, 5) and thus have not been fully characterized.

Ciliary neurotrophic factor (CNTF) has been shown to be a potent neuroprotective cytokine in various animal models of retinal degeneration (6). Owing to its broad-spectrum trophic effects, a number of CNTF clinical trials have been conducted, including those aimed at treating RP (7–13). However, with the exception of trials for macular telangiectasia type 2 (9, 14), most other trials have not shown significant efficacy for CNTF treatments. We have simulated clinical trial scenarios by expressing the same secreted recombinant CNTF used in human trials in a mouse model for dominant RP due to the Perpherin2/Prph2 P216L mutation (15). CNTF primarily triggers the JAK-STAT and ERK signaling pathways in the mouse retina (15, 16). Despite enhancing photoreceptor viability, constitutive CNTF signaling alters the retinal transcriptome and suppresses visual function (15, 17). Molecular genetic analysis has demonstrated that exogenous CNTF signals are transmitted by the cytokine receptor gp130 to initially activate STAT3 and ERK in Müller glial cells and subsequently induce cytokine signaling in rods to promote photoreceptor survival (18). Further mechanistic investigations revealed that exogenous CNTF treatment profoundly impacts the metabolism of the degenerating retina, leading to enhanced anabolic activity, increased energy supply, and restored redox capacity to promote neuronal viability (19).

As a member of the suppressor of cytokine signaling (SOCS) family, SOCS3 is a direct target gene of tyrosine-phosphorylated STAT3, which can dimerize and enter the nucleus to regulate transcription (20–22). Once induced in response to cytokine signaling, SOCS3 acts as a negative feedback inhibitor by binding to the complex of the cytokine receptor and its associated JAK kinase, either hampering JAK activation or mediating the ubiquitination and subsequent proteasome degradation of cytokine receptors (23–25). In the developing retina, CNTF signaling can suppress rod photoreceptor differentiation (16, 26), and deletion of SOCS3 has been implicated in regulating the temporal onset of rhodopsin gene expression (27). In the optic nerve crush mouse model, SOCS3 deletion has been shown to promote the regeneration of injured retinal ganglion cell axons (28). Consistent with the influence of CNTF on retinal metabolism (19), the codeletion of SOCS3 and PTEN in adult retinal ganglion cells promotes not only the growth of injured axons but also the formation of functional synapses with suprachiasmatic neurons (29, 30). In the adult retina, ocular inflammation and high levels of glucose caused retinal endothelial cell stress can both induced SOCS3 (31) (32), indicating a response to retinal endogenous cytokine releases.

Since cytokine receptor gp130-mediated CNTF signaling is required in rod cells to result in STAT3 activation and neuroprotection (18), we hypothesize that removing SOCS3 from rods may increase endogenous JAK-STAT3 signaling to promote photoreceptor survival without exogenous CNTF treatment. In this study, we tested this hypothesis by ablating SOCS3 in rod cells in two mouse RP models with different degeneration rates. Our results show that ablation of SOCS3 in rod photoreceptors elicits broad STAT3 and ERK activation among different retinal cells, and is sufficient to attenuate rod degeneration and improve cone morphology and survival without the delivery of exogenous CNTF. In addition, the rod-specific deletion of SOCS3 led to partial visual function rescue in the fast degeneration RP model and sustained cone function in the slow degeneration RP model. These findings provide insight into the regulatory mechanism controlling endogenous retinal signaling and point to new therapeutic strategies.

## RESULTS

### Rod-specific SOCS3 deletion attenuates photoreceptor degeneration

**Figure 1:**
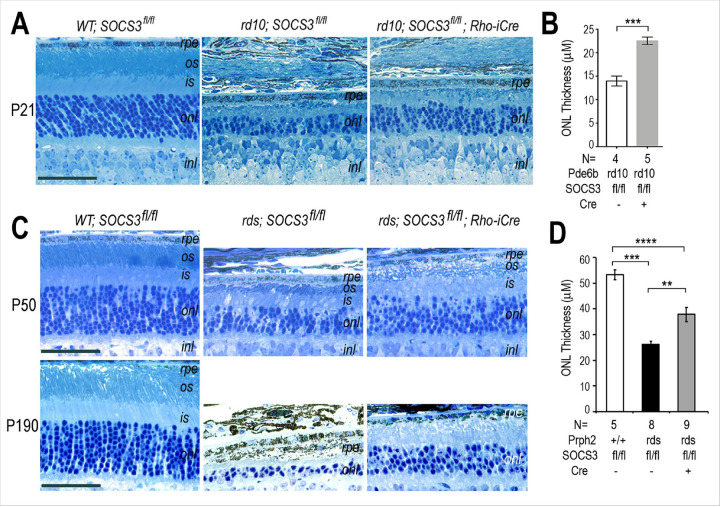
**A.** Microscopy images showing the outer retinas of the WT and rd10 mutant in the SOCS3 homozygous conditional allele (SOCS3^fl/fl^) background with or without Rho-iCre at P26. **B.** Bar graph showing measurements of ONL thickness in the rd10 background at P26. The genotypes and numbers of independent animals measured are indicated below. Statistically significant *p* values are indicated, *** p<0.001. **C.** Microscopy images showing the outer retinas of the WT and rds mutant in the SOCS3 homozygous conditional allele (SOCS3^fl/fl^) background with or without Rho-iCre at P50 and P190. **D.** Bar graph showing the ONL thickness of WT and rds retinas at P52. The genotypes and numbers of independent animals measured are shown below. Significant *p* values are indicated, ** p<0.01, *** p<0.001, **** p<0.0001. Scale bars for all, 50 mm. *rpe*, retinal pigment epithelium; *os*, outer segment; *is*, inner segment; *onl*, outer nuclear layer; *inl*, inner nuclear layer. Effects of rod-specific SOCS3 deletion on photoreceptor survival.

We examined whether rod-specific SOCS3 deletion could protect photoreceptors from degeneration in rd10 mice harboring a homozygous Pde6b gene mutation (33), which leads to rapid photoreceptor degeneration within the first postnatal month. The rod-specific Rho-iCre (34) was introduced into the double homozygous rd10 and conditional SOCS3 allele (35) background. Histological examination revealed severe outer nuclear layer (ONL) thinning by more than 50% in rd10 mice at postnatal day 21 (P21), with shortened inner and outer segments compared with those of the wild-type control (Fig. 1A). Rod-specific SOCS3 deletion (referred to herein as SOCS3 rod KO) in the rd10 background resulted in a thicker ONL than in the rd10 background, indicating attenuation of photoreceptor loss. Morphometric quantification confirmed significant panretinal rescue, with the ONL thickness of SOCS3 rod-KO retinas at 22.52 ± 0.80 μm and that of the rd10 retina at 13.96 ± 1.06 μm at P26 (Fig. 1B).

We next performed rod-specific deletion of SOCS3 in a dominant RP model, which carries the mutant Prph2(P216L) transgene to cause relatively slow degeneration (36). Compared with the wild-type controls, the Prph2(P216L)/rds retinas (referred to herein as rds) presented approximately 50% thinning of the ONL at P50, whereas SOCS3 rod KO resulted in an increase in ONL thickness, indicating increased photoreceptor survival (Fig. 1C). Morphometric quantification revealed that at P52, the retinas of the wild-type, rds, and rds with SOCS3 rod KO strains presented ONL thicknesses of 53.33 ± 1.89 μm, 26.15 ± 1.29 μm, and 37.83 ± 2.7 μm, respectively (Fig. 1D). The rescuing effect became more prominent at P190, when the rds retina showed a severe loss of photoreceptors, whereas the rds retina with SOCS3 rod KO retained a substantial ONL similar to that of the P52 retina (Fig. 1C). Together, the results of the histological analysis demonstrated that rod-specific SOCS3 deletion prolonged the survival of photoreceptors in two RP models in which distinct mutations caused degeneration.

### SOCS3 rod KO improves photoreceptor morphology and survival

**Figure 2:**
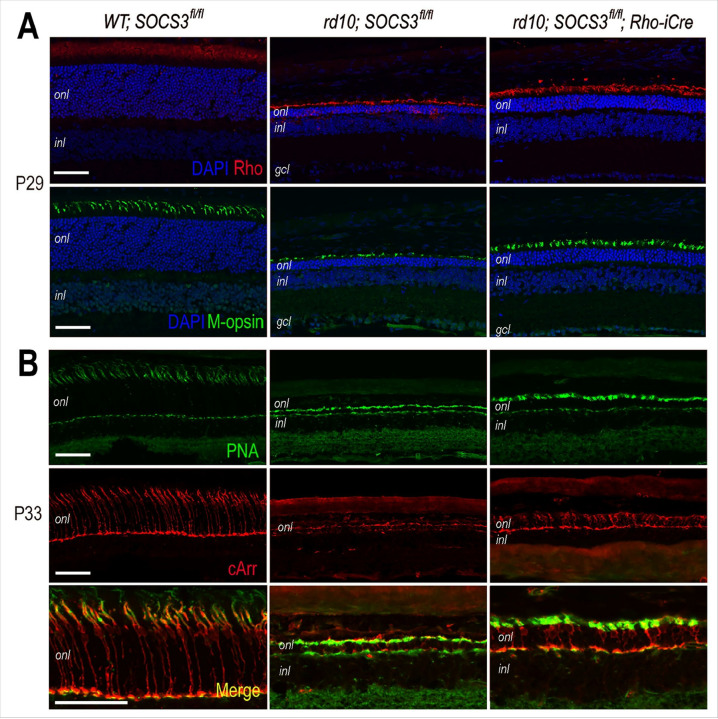
Influence of rod-specific *SOCS3* deletion on photoreceptor marker expression in the rd10 retina. **A.** Confocal images of merged DAPI and immunofluorescent signals of rhodopsin (Rho) and m-opsin in WT and rd10 mutants with or without Rho-iCre at P29. Deletion of SOCS3 in rods corrects Rho mislocalization. **B.** Confocal images showing immunolabeling for cone arrestin (cArr) and peanut agglutinin (PNA) in WT and rd10 mutants with or without Rho-iCre at P33. Deletion of SOCS3 in rods improved cone morphology. *onl*, outer nuclear layer; *inl*, inner nuclear layer; *gcl*, ganglion cell layer. Scale bars, 50 mm.

We previously reported that lentiviral vector-mediated expression of recombinant human CNTF corrected the mislocalization of rhodopsin and cone opsin found in the Prph2(P216L)/rds retina (18). Therefore, we examined whether SOCS3 rod KO had similar effects. Anti-rhodopsin immunolabeling and confocal imaging detected ectopic rhodopsin in the retina at P29, whereas the SOCS3 rod-KO rd10 retinas presented longer outer segments with corrected rhodopsin localization (Fig. 2A). Furthermore, SOCS3 rod KO improved cone outer segments, as revealed by the m-opsin distribution pattern (Fig. 2A). Further evaluation of cone photoreceptor status by immunolabeling for two additional markers, cone arrestin (cArr) and peanut agglutinin (PNA), revealed improved cone morphology at P33 (Fig. 2B). Compared with those in wild-type retinas, cone cells in rd10 retinas presented diminished cArr expression and collapsed PNA labeling, whereas SOCS3 rod KO resulted in more robust cone cell soma and cone pedicles (Fig. 2B).

**Figure 3:**
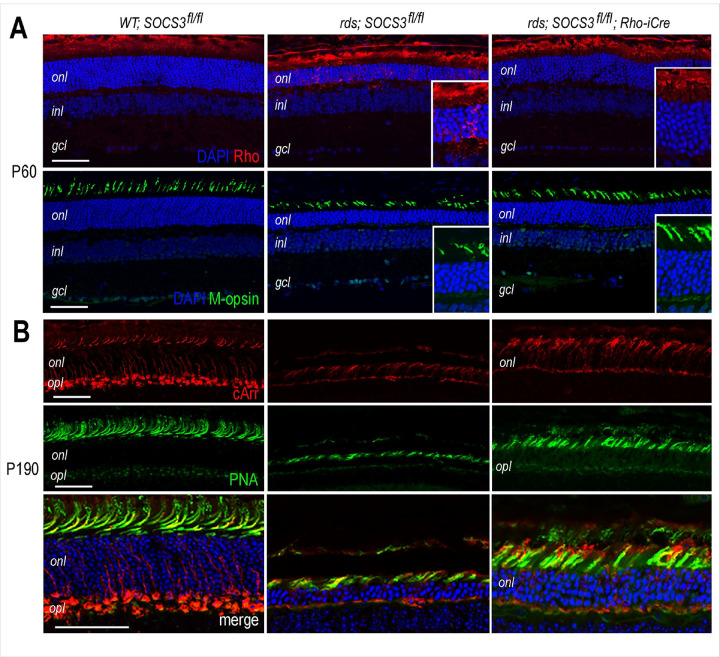
Influence of rod-specific SOCS3 deletion on photoreceptor marker expression in the rds retina. **A.** Confocal images of merged DAPI and immunofluorescent signals of rhodopsin (Rho) and m-opsin in WT and rds mutants with or without Rho-iCre at P60. Deletion of SOCS3 in rods corrects Rho mislocalization. Insets are magnified 2-fold. **B.** Confocal images showing immunolabeling for cone arrestin (cArr) and peanut agglutinin (PNA) in WT and rds mutants with or without Rho-iCre at P190. Deletion of SOCS3 in rods preserves cone morphology. *onl*, outer nuclear layer; *inl*, inner nuclear layer; *gcl*, ganglion cell layer. Scale bars, 50 mm.

Similar immunocytochemistry analysis of the rds retinas revealed that SOCS3 rod KO corrected rhodopsin mislocalization and lengthened inner and outer segments, as indicated by rhodopsin and mopsin labeling at P60 (Fig. 3A). The improved cone survival in rds retinas with SOCS3 rod KO persisted past 6 months, as demonstrated by cArr and PNA labeling at P190 (Fig. 3B). However, although SOCS3 rod KO improved cone outer segment and soma morphology, the cone pedicles in the rds retina were not as well preserved in comparison with those in the wild-type retina (Fig. 3B).

**Figure 4:**
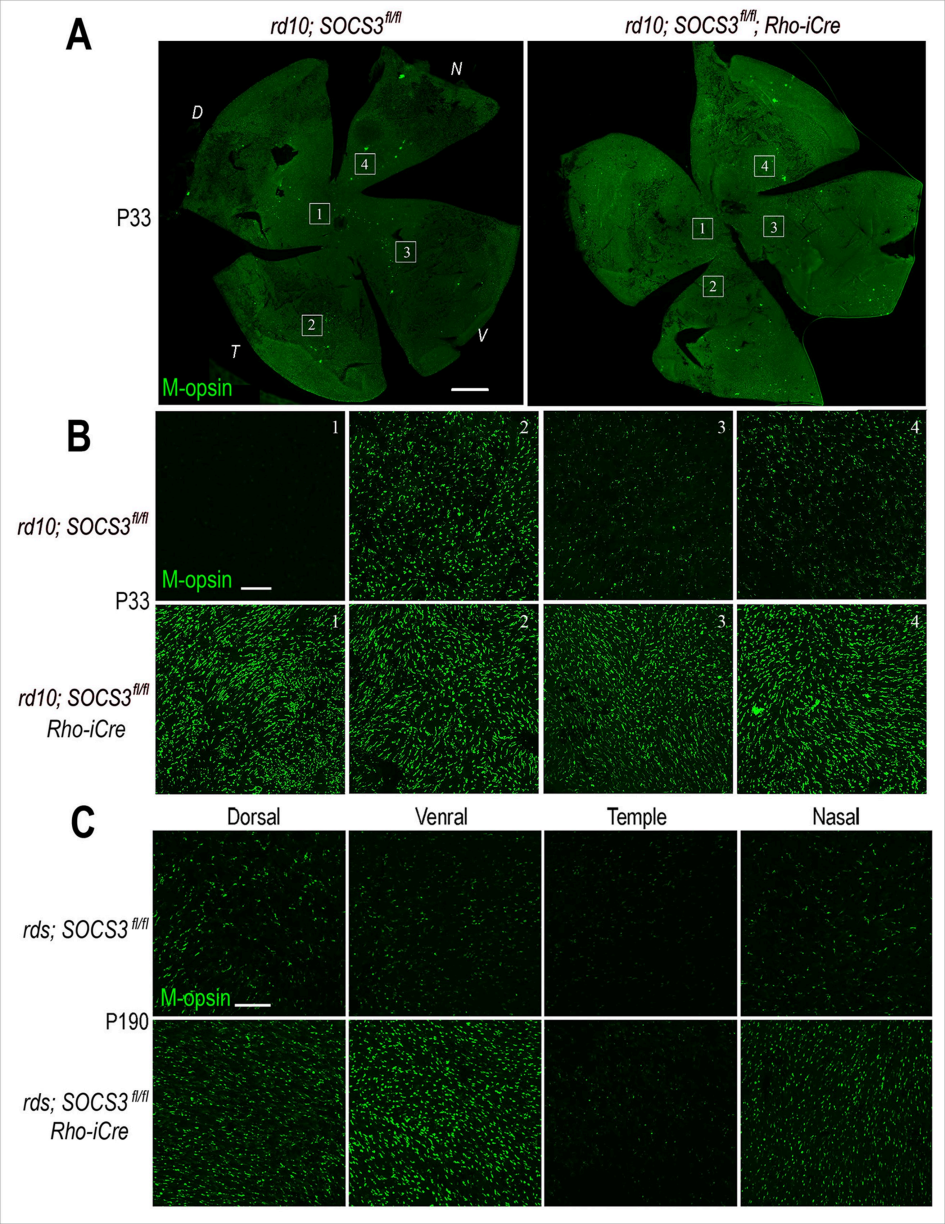
Effects of rod-specific SOCS3 deletion on cone photoreceptor cell survival. **A.** Confocal images of flat-mounted rd10 retinas with or without Rho-iCre labeled for m-opsin at P33. *D*, dorsal; *V*, ventral; *N*, nasal; *T*, temple. Scale bars, 500 mm. **B.** Higher magnification images from different regions of the P33 rd10 retinas shown in A are labeled with numbers. Scale bars, 50 mm. **C**. Confocal images of m-opsin labeling from different regions of flat-mounted rds retinas with or without Rho-iCre at P190. Scale bars, 50 mm.

To further assess the effect of SOCS3 rod KO on cone survival in rd10 and rds mice, we imaged flat-mounted retinas labeled with m-opsin. The rd10 retinas presented reduced cone density in the central retina at P33; SOCS3 rod KO significantly increased the number of m-opsin-positive cones in the central retina (Fig. 4A, B). Similarly, the rds retinas with SOCS3 rod KO showed increased cone viability at P190 compared with rds alone (Fig. 4C).

### SOCS3 rod KO activates panretinal STAT3 and ERK signaling

**Figure 5:**
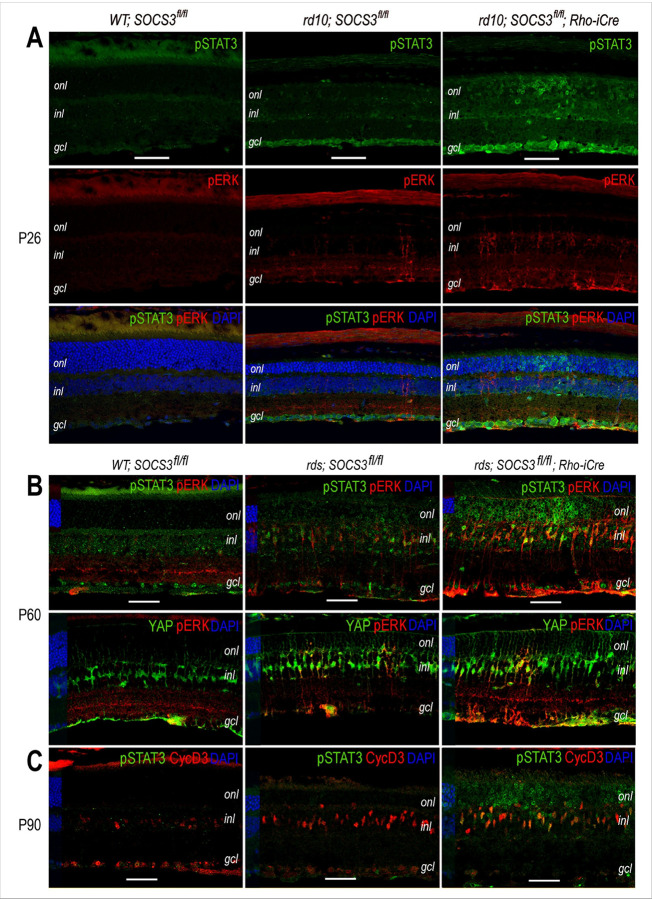
Signaling events induced by rod-specific SOCS3 deletion in rd10 and rds mutant retinas. **A.** Confocal images of immunofluorescent signals for phospho-STAT3 (pSTAT3) and phospho-ERK1/2 (pERK) in WT and rd10 retinas with or without Rho-iCre at P26. **B.** Confocal images of co-immunolabeling for pSTAT3 and pERK and for YAP and pERK in WT and rds retinas with or without Rho-iCre at P60. **C.** Confocal images of co-immunolabeling signals for pSTAT3 and CycD3 in WT and rds retinas with or without Rho-iCre at P90. Scale bars, 50 mm. *onl*, outer nuclear layer; *inl*, inner nuclear layer; *gcl*, ganglion cell layer.

Exogenous CNTF induces STAT3 and ERK activation in degenerating rds retinas (15, 18). To determine whether SOCS3 rod KO could trigger similar cellular signaling events, we performed immunolabeling and confocal imaging to detect phosphorylated signaling molecules. Immunocytochemistry revealed low levels of tyrosine-phosphorylated STAT3 (pSTAT3) and increased sporadic phosphorylation of p42/44 ERK (pERK) in the retina of rd10 compared with the wild-type control (Fig. 5A), indicating that the Pde6b mutation caused low intrinsic signaling events. In the rd10 retinas of SOCS3 rod KO mice, pSTAT3 and pERK signals were detected throughout the entire retina. In the ONL, the typical ring-like pSTAT3 labeling pattern for rod euchromatin was detected as expected. However, pSTAT3 signals were not limited to the ONL but were also detected in the inner nuclear layer (INL) and increased in the ganglion cell layer (GCL) (Fig. 5A). Similarly, rd10 retinas with SOCS3 rod KO presented panretinal ERK activation in the INL and GCL.

Compared with those in the control retina, the rds retinas at P60 presented low levels of pSTAT3 in a subset of rod cells with a ring-like labeling pattern (Fig. 5B). In rds retinas with SOCS3 rod KO, STAT3 activation in rod cells was significantly intensified in the ONL as well as in a subset of INL cells at P60 (Fig. 5B). In addition, ERK activation was also increased by SOCS3 rod KO (Fig. 5B). The pERK signals in the SOCS3 rod KO retina were mostly distributed in the INL, the inner plexiform layer (IPL), and the ganglion fiber layer (Fig. 5B). Colabeling with the Müller glial marker YAP confirmed that ERK activation was prominent in a subset of Müller glial cells (Fig. 5B). Furthermore, colabeling with the adult Müller glial marker Cyclin D3 (CycD3) at P90 confirmed that most pSTAT3-positive INL cells activated by SOCS3 rod KO were Müller glia. These analyses of signaling molecules in the rd10 and rds retinas demonstrated that rod-specific SOCS3 KO not only elicited STAT activation in rod photoreceptors but also led to signal propagation toward the inner retina, especially by inducing the activation of both STAT3 and ERK in Müller glia.

### SOCS3 rod KO improves the visual function of degenerating retinas

**Figure 6:**
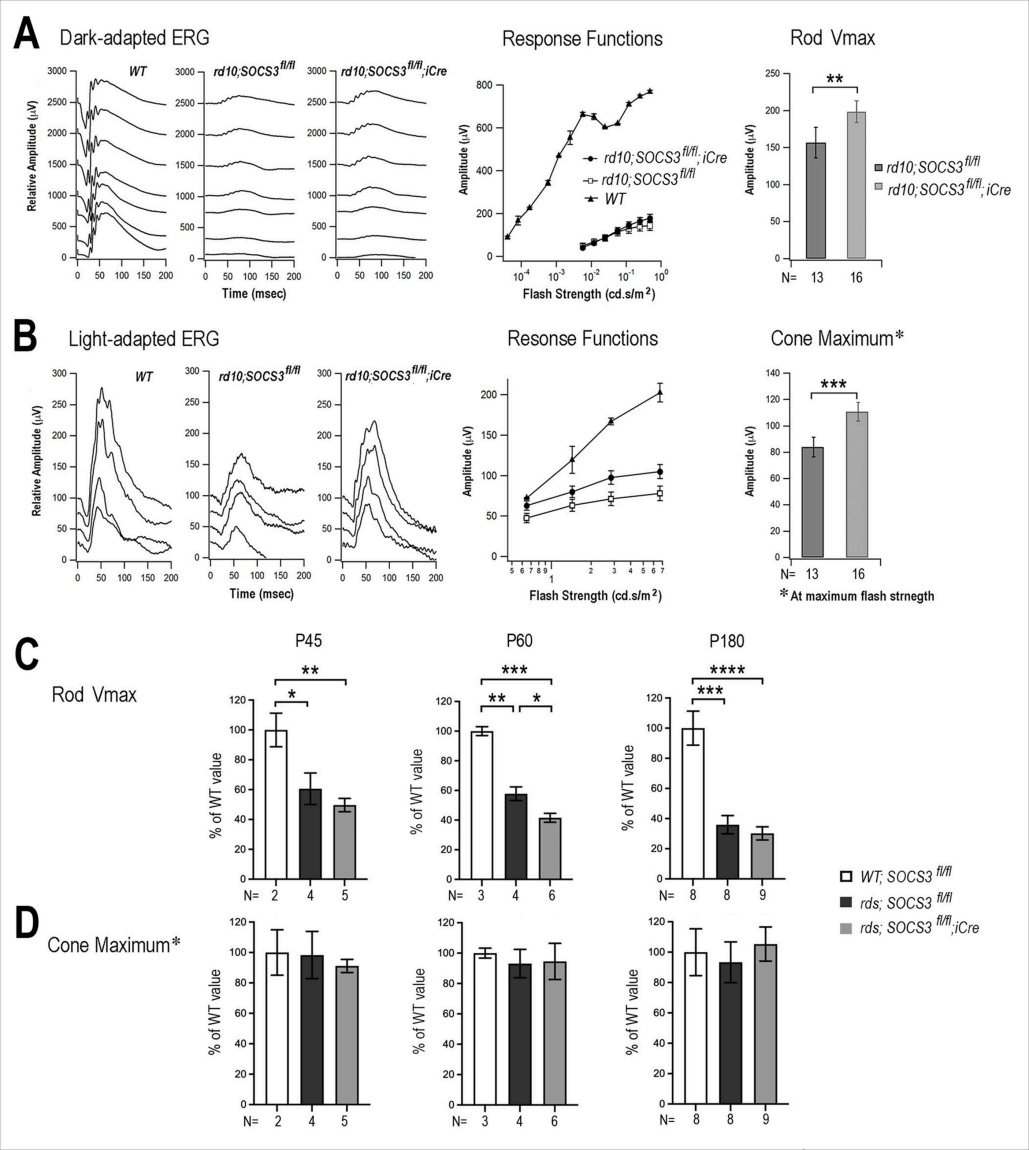
Impact of rod-specific SOCS3 deletion on the visual function of the rd10 and rds mutants. ERG results for WT and rd10 mice at P39 with or without SOCS3 rod deletion are presented. **A.**Dark-adapted ERGs for rd10 showing representative tracings, response amplitudes as a function of light flash intensity, and the rod response Vmax. **B.** Light-adapted ERGs for rd10 showing representative tracings, response amplitudes as a function of light flash intensity, and the rd10 cone response at maximum light illumination. ERG results for WT and rds mice with or without SOCS3 rod deletion at P45, P60, and P180 are presented in C and D: **C.** Bar graphs showing the rod response Vmax. **D.**Bar graphs showing the cone response under maximum light illumination. The numbers of independent animals (N) recorded are indicated below the bar graphs. Significant *p* values are indicated, * p<0.05, ** p<0.01, *** p<0.001, **** p<0.0001.

To determine whether SOCS3 rod KO-enhanced photoreceptor survival impacts visual function, we performed electroretinography (ERG). Compared with the wild-type controls, the dark-adapted a-wave amplitudes of the rd10 mice were diminished at all the different light flash intensities by P39, reflecting the severe loss of rod cells (Fig. 6A). However, with increasing flash intensities, the rd10 mice with SOCS3 rod KO presented greater dark-adapted amplitudes than did the rd10 mice. We also used V_max_, the saturated maximal amplitude, obtained from a fit of the Naka–Rushton function to the dark-adapted ERGs, to assess rod functions. The V_max_ for the SOCS3 rod KO rd10 eyes was 198.4 ± 14.57 μV, whereas the corresponding V_max_ for the rd10 eyes was 156.5 ± 20.73 μV, demonstrating a significant improvement in the a-wave (Fig. 6A). With respect to light-adapted ERG responses, SOCS3 rod KO mice also presented greater amplitudes than did the rd10 mice at P39 (Fig. 6B). At the brightest stimulus intensity, the cone maximum response value for SOCS3 rod KO was 110.7 ± 7.185 μV, whereas that for the rd10 control was 83.96 ± 7.480 μV (Fig. 6B). These significant improvements in both rod and cone functions were consistent with the enhanced photoreceptor survival in the rd10 mice caused by rod-specific deletion of SOCS3.

To assess the impact of SOCS3 rod KO on visual function in rds mice with a slow rate of degeneration, ERG assays were performed over a 6-month period. Analysis of the dark-adapted ERGs at P45 revealed that the V_max_ values for the rds eyes and rds with SOCS3 KO were significantly reduced to 60.6 ± 10.5% and 50.7 ± 4.5% of the wild-type control level, respectively (Fig. 6C). By 6 months, the V_max_ values for rds and rds with SOCS3 KO were further reduced to 35.9 ± 6.1% and 30.2 ± 4.3% that of the wild type, respectively (Fig. 6C). In contrast to the continued decrease in the dark-adapted ERGs reflecting rod degeneration, the cone maximum values in the rds retina did not significantly decrease from P45 to P180 (Fig. 6D). Moreover, in rds eyes, SOCS3 rod KO did not reduce the cone maximum compared with rds alone, indicating sustained maintenance of light-adapted visual function over the 6-month period (Fig. 6D). These results demonstrated that unlike ERG suppression by exogenous CNTF treatment, rodspecific deletion of SOCS3 in the rds retina did not cause detrimental loss of cone function.

### SOCS3 rod KO has differential effects on retinal gene transcription

**Figure 7:**
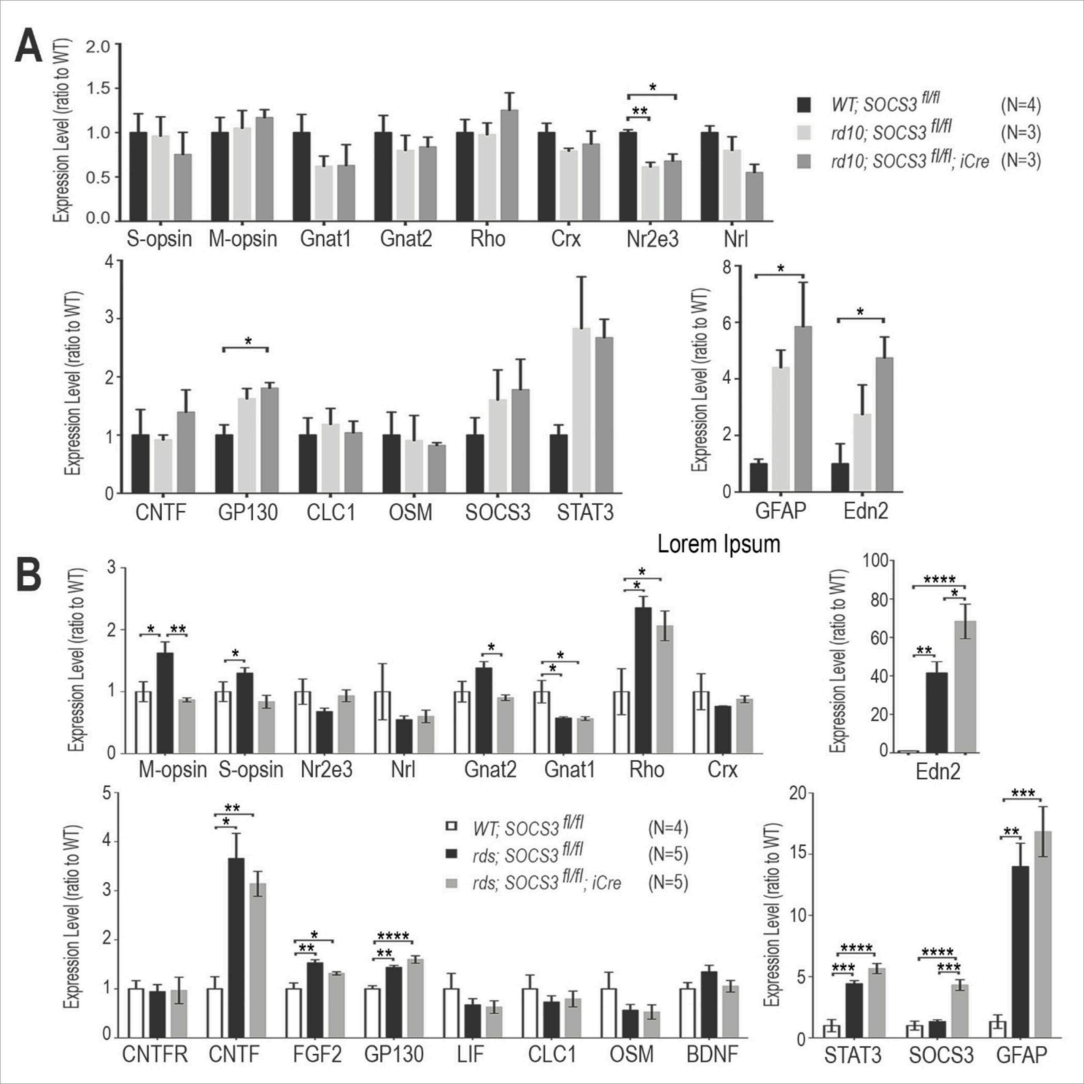
Influence of rod-specific SOCS3 deletion on transcript levels in rd10 and rds mutants. Quantitative PCR results with or without rod-specific SOCS3 deletion are shown. **A**. Bar graphs showing gene expression levels detected by qPCR in the rd10 retina at P24. **B**. Bar graphs showing gene expression levels measured by qPCR in therds retinas at P60. The transcript levels are presented as ratios to those in the WT retina. The numbers of independent samples (N) are indicated for each mutant and WT retina. Genes with significantly altered expression are indicated. * p<0.05, ** p<0.01, *** p<0.001, ****, p<0.0001.

To determine whether SOCS3 rod KO-triggered signaling events influence retinal gene expression, we used quantitative RT‒PCR to measure a set of selected genes previously shown to respond to CNTF treatment (17, 18). At P24, the rd10 mutant retina contained increased transcript levels of glial fibrillary acidic protein (GFAP) and endothelin 2 (Edn2) (Fig. 7A), both of which are known to be induced in response to photoreceptor degeneration (37, 38). SOCS3 rod KO in the retina of rd10 mice caused only slight increases in GFAP and Edn2 (Fig. 7A). Compared with the wild-type retina, the cytokine receptor gp130 and STAT3 were elevated in the rd10 retina (Fig. 7A), but SOCS3 rod KO did not affect their expression further. Moreover, rod-specific SOCS3 deletion did not significantly influence the transcript levels of the photoreceptor transcription factors Crx and Nrl, the photopigment genes rhodopsin and opsins, or the transducin subunits Gnat1 for rods and Gnat2 for cones (Fig. 7A). The transcript of the nuclear hormone receptor Nr2e3 was downregulated in the retina of rd10 but was not further affected by SOCS3 rod deletion (Fig. 7A). These results suggested that in rapidly degenerating rd10 retinas, SOCS3 rod KO had a limited effect on the expression of these selected genes.

We next analyzed gene expression status in the retinas of the rds at P60 via quantitative RT‒PCR. In contrast to the rd10 retina, the rds mutant retina presented 40-fold higher Edn2 and 14-fold higher GFAP levels than the wild-type retina did, and SOCS3 rod KO caused significant further increases in both Edn2 and GFAP (Fig. 7B). The rds retinas also presented elevated transcript levels of CNTF and FGF2, as well as the cytokine receptor gp130 and the effector STAT3 (Fig. 7B). However, the transcript levels of these signaling molecules were not affected by SOCS3 rod KO. The exception was the SOCS3 transcript itself, which was significantly elevated in the retina of rds with SOCS3 rod deletion, likely reflecting a response to the overall elevation of cytokine signals in the rds retina. In addition, the rds retina presented altered expression of several photoreceptor-specific genes compared with the wild-type control retina (Fig. 7B). Notably, SOCS3 rod KO in the rds retina resulted in a reduction in both m-opsin and Gnat2 in cone cells (Fig. 7B). These results suggested that rod ablation of SOCS3 in the rds retina had a stronger cumulative effect on gene expression than ablation in the rd10 retina.

## DISCUSSION

In this study, we performed rod-specific SOCS3 deletion in two preclinical mouse models of RP to investigate whether manipulating endogenous Jak-STAT3 signaling affects neuronal viability. Our results validated the hypothesis that eliminating the cytokine signaling inhibitor SOCS3 function in rods is sufficient to attenuate photoreceptor loss without the supply of exogenous CNTF. Furthermore, the loss of SOCS3 function in rods elicits panretina activation of STAT3 and ERK and results in partial preservation of visual function in RP model mice. These findings provide insight into the regulatory mechanisms of intrinsic signaling events in the degenerating retina and suggest potential strategies to achieve both morphological and functional rescue.

Both RP models exhibit photoreceptor gene mutations but with distinct degeneration rates. rd10 carries a recessive mutation in the Pde6b gene that is required for rod phototransduction and that results in rapid degeneration (33). The rds mouse, on the other hand, carries the dominant P216L mutation in Prph2, which is involved in rod and cone outer segment disc morphogenesis (36, 39, 40). The deletion of SOCS3 in the rd10 and rds retinas similarly enhances rod and cone viability; however, owing to the different rates of degeneration, the lengths of the survival periods are dependent on the particular gene mutation causing cell death. In both the rd10 and rds retinas, immunolabeling revealed panretinal activation of STAT3 and ERK due to SOCS3 rod KO, but the slowly degenerating rds retina experienced a longer period of exposure to elevated pSTAT3 signaling. Thus, in contrast to the rd10 retina, where SOCS3 rod KO causes minimum phototransduction gene perturbation, the rds retina shows stronger transcription perturbations, including increased levels of endogenous CNTF and FGF2, as well as alterations in phototransduction genes. These findings suggest that a particular RP-causing mutation may influence endogenous signaling activities and therapeutic outcomes.

In addition to the different periods of neuronal survival, SOCS3 rod deletion in the rd10 model clearly improved both rod and cone visual function. In contrast, rds retinas with SOCS3 rod KO showed a slow decline of rod V_max_ over time. Strikingly, SOCS3 rod KO in the rds retina did not impact cone function over a period of 6 months. This result is especially noteworthy since although exogenous CNTF effectively prevents photoreceptor death, the treatment also leads to severe suppression of visual function, rendering the treatment ineffective. In the case of AAV-mediated CNTF expression driven by the strong CAG promoter, the scotopic and photopic b-waves in the rds mouse at P70 are suppressed by an average of 69% and 71%, respectively (15). By P90, scotopic and photopic b-waves further decrease by 81 to 83% despite photoreceptor survival (15). This detrimental visual function suppression is likely due to constitutively high levels of STAT3 activation, which modifies the retinal transcriptome, including the downregulation of phototransduction genes (15, 17). The improvement of visual function in the rapid degenerating rd10 retina and the maintenance of cone function in the rds retina are likely due to the relatively low levels of cytokine signaling induced by SOCS3 rod KO, which does not significantly alter the transcriptome. These results suggest that controlling the intensity and duration of CNTF downstream signaling events is critical for achieving neuroprotection and preserving function.

Increasing evidence has indicated that photoreceptor degeneration causes retinal network remodeling, including microglial activation (41) and Müller glial cell responses (42). In the rds retina, exogenous CNTF initially induces the phosphorylation of STAT3 and ERK in Müller glia and subsequently activates STAT3 in rod cells (18). It is therefore anticipated that rod-specific SOCS3 deletion would increase pSTAT3 in rod photoreceptors, but SOCS3 deletion-induced STAT3 activation is not limited to rods but also propagates to Müller glial cells. In addition, pERK signals are significantly intensified in Müller glial processes and end feet in SOCS3 rod-KO retinas. These data support an intricate signaling loop between photoreceptors and Müller glia, which is dynamically involved in retinal homeostasis and neuronal survival.

Rod-specific SOCS3 deletion not only attenuates rod cell loss but also leads to improved cone cell morphology and viability. This is not unexpected, as the dependency of cone survival on rods has been well documented (43, 44). Accumulating evidence suggests that cell‒cell signaling and metabolic regulation play important roles in rod and cone photoreceptor survival under degeneration conditions (45–50). We have shown that CNTF-mediated neuroprotection involves a profound impact on the metabolic status of degenerating retinas, including enhancing glycolytic and anabolic metabolism, increasing the energy supply, and restoring redox capacity (19). Whether the activation of cytokine signaling influences molecular exchanges, including metabolite exchanges among retinal neurons and glia, remains to be further investigated.

In summary, our findings in preclinical models of inherited retinal degeneration show that antagonizing the cytokine signaling inhibitor SOCS3 can potentiate endogenous neuroprotective capacities and effectively prolong neuron survival without compromising visual function. These results also highlight the importance of regulating signaling intensity and duration of exogenous neuroprotective agents to avoid detrimental effects and suggest a new therapeutic strategy by eliciting endogenous neuronal surviving potential.

## MATERIALS AND METHODS

### Animals and genotyping

The rd10 mouse with a recessive missense mutation in the Pde6b gene exon 13 (33) (JAX stock number 004297) and the SOCS3 mouse carrying a conditional allele (35) (JAX stock number 010944) were purchased from the Jackson Laboratory (Bar Harbor, ME) and backcrossed into the C57BL/6J background. The rds mouse carrying the Prph2(P216) transgene (36) was obtained from Dr. Gabriel Travis and maintained on the wild-type Prph2^+/+^ background. To perform rod-specific SOCS3 deletion in the rd10 mouse, genetic crosses with the mouse carrying the transgene Rho-iCre75 (34) (gift from Dr. Jason C.-K. Chen) were carried out to generate double homozygous SOCS3^flox/flox^;Pde6b^rd10/rd10^ mice either with or without Rho-iCre. To perform rod-specific SOCS3 deletion in Prph2(P216L)/rds mice, genetic crosses with Rho-iCre mice were carried out to generate SOCS3^flox/flox^;Prph2^(P216L)/+^ mice with or without Rho-iCre. Age-matched wild-type mice carrying SOCS3^flox/flox^ were also used as controls. PCR genotyping was carried out using genomic DNA extracted from tail biopsy tissues and the PCR primers listed in Table S1.

The use of animals and all experimental procedures with animals were approved by the Animal Research Committee of the University of California Los Angeles and were performed in compliance with the National Institutes of Health Guide for the Care and Use of Animals and The Association for Research in Vision and Ophthalmology Statement for the Use of Animals in Ophthalmic and Vision Research.

### Histology and morphometrics

Histological semithin sections of 1 μm thickness were prepared by fixing mouse eyes in 2% (wt/vol) formaldehyde and 2.5% (wt/vol) glutaraldehyde in 0.1 M sodium phosphate buffer and processed as described previously (51). The sections were counterstained with toluidine blue, and bright field images were captured. To measure the thickness of the outer nuclear layer (ONL), 14 μm cryosections of eyes fixed with 4% (wt/vol) paraformaldehyde in PBS were stained with DAPI. A minimum of three central sections containing the optic nerve head from each eye were used to acquire digital images of the retina. The thickness of the ONL demarcated by DAPI-positive photoreceptor nuclei was measured on both sides at positions 200 μm away from the optic nerve head.

### Immunofluorescent labeling and confocal imaging

For immunofluorescent labeling with antibodies, the tissues were fixed with 4% (wt/vol) paraformaldehyde in PBS and processed as described previously (15). Whole-mount retinas were incubated overnight with primary and secondary antibodies at 4°C, followed by extensive washes.

Fluorescence images were captured via an Olympus FluoView 1000 confocal microscope. The antibodies used are summarized in Table S2.

### Quantitative PCR

Total RNA was extracted from mouse retinas via an RNeasy Mini Kit (Qiagen). First-strand cDNA was synthesized with the SuperScript III FirstStrand Synthesis System (Thermo Fisher Scientific). PCR was carried out with SYBR Green PCR Master Mix (Applied Biosystems/Life Technologies, USA) in a total volume of 10 μl using the primers for real-time PCR listed in Table S1. A Light Cycler 480 II (Roche Applied Science, Mannheim, Germany) instrument was used for amplification and real-time quantitative detection of the PCR products. The target gene expression levels were normalized to the threshold cycle (Ct) of mouse *GAPDH*. The expression level of each gene was calculated relative to the expression of the control group: 2-ΔΔCt, where ΔΔCt = Exp (Ct, target ± Ct, GAPDH) ± Ctrl (Ct, target ± Ct, GAPDH). The data are shown as the mean ± SEM of three replicates.

### Electroretinogram

Following overnight dark adaptation, the mice were anesthetized via an intraperitoneal injection of saline containing ketamine (150 mg/kg body weight) and xylazine (5 mg/kg body weight). Electroretinograms (ERGs) were recorded from the corneal surface after pupil dilation (1% atropine sulfate) via a gold loop corneal electrode together with a mouth reference and a tail ground electrode as described previously (5). A drop of methylcellulose (2.5%, wt/vol) on the corneal surface was used to ensure electrical contact and to maintain corneal integrity. Body temperature was maintained at 38°C with a heated water pad. All stimuli were presented in a large integrating sphere coated with highly reflective white matte paint (#6080; Eastman Kodak Corp.). The responses were amplified (Grass CP511 AC amplifier, ×10,000) and digitized via a data acquisition board (PCI-1200; National Instruments) on a personal computer. Signal processing was performed with custom software (LabWindows/CVI; National Instruments). For each stimulus condition, the responses were computer-averaged, with up to 50 records averaged for the weakest signals. A signal rejection window was adjusted online to eliminate artifacts. Dark-adapted ERGs were recorded as blue (Kodak Wratten 47A) light flashes up to a maximum intensity of 0.42 cd.s/m^2^. Cone-mediated responses were obtained with white flashes up to a maximum of 4.35 cd.s/m^2^ on a rod-saturating background (32 cd.s/m^2^). All stimuli were presented at 1 Hz except for the brightest flashes, where the presentation rate was slowed to 0.2 Hz (52).

#### Statistical analysis

Statistical analysis was performed via Student's t tests or two-way ANOVA when appropriate. All experimental values are expressed as the means ± SEs, and P < 0.05 was considered statistically significant. For ONL thickness quantification between two conditions, an unpaired Student's t test was used. For the visual functional test, two-way ANOVA was used to analyze the mean difference between the two groups. The results were analyzed using Prism 8.0 by GraphPad.

## Supplementary Material

Supplementary Files

This is a list of supplementary files associated with this preprint. Click to download.

- TableS1S2.docx

## SIGNIFICANCE

The cytokine CNTF has been tested in clinical trials and approved by the FDA to treat major retinal degenerative diseases on the basis of its high neuroprotective potency in preclinical models. However, persistent CNTF treatment has been shown to alter retinal gene expression and cause visual function decline. Here, we provide evidence that eliminating a major cytokine inhibitor from rod cells is sufficient to increase endogenous cellular signaling events and prolong photoreceptor survival without exogenous CNTF. Moreover, this approach results in minimal detrimental effects compared with CNTF treatments in two preclinical models of retinitis pigmentosa. These findings support modulating innate neuroprotective signals as an effective strategy for treating various retinal degenerative diseases.
